# Mucormycosis Leading to Cerebral Edema and Cerebellar Tonsillar Herniation after Allogeneic Bone Marrow Transplant: A Case Report

**DOI:** 10.1155/2019/5138198

**Published:** 2019-11-11

**Authors:** Chetan Jeurkar, Lauren Margetich, Ziver Sahin

**Affiliations:** ^1^Thomas Jefferson University Hospital, Department of Medical Oncology, Philadelphia, PA, USA; ^2^Sidney Kimmel Medical College, Philadelphia, PA, USA; ^3^Thomas Jefferson University Hospital, Department of Pathology, Philadelphia, PA, USA

## Abstract

**Introduction:**

Mucormycosis following hematopoietic stem cell transplant (HSCT) carries a very high mortality rate. Pulmonary mucormycosis often leads to systemic dissemination and eventual death. It is imperative for transplant providers to have a high level of suspicion for mucormycosis and initiate early treatment. Here, we present a 64-year-old woman who died of disseminated mucormycosis 13 days following her allogeneic HSCT.

**Case Presentation:**

A 64-year-old female with a history of acute myeloid leukemia (AML) presented for allogeneic HSCT and passed away from intracerebral hemorrhage secondary to mucormycosis infection 13 days following her transplant. On autopsy, it was found she had angioinvasive mucormycosis in her frontal lobe leading to cerebral edema which eventually led to tonsillar herniation and brainstem infarction. Her lungs were the likely source of infectious dissemination.

**Discussion:**

This case represents an unusual course of events following HSCT in that no other published case shows tonsillar herniation resulting from mucormycosis-related intracerebral swelling. We also report this case because it is believed mucormycosis in HSCT patients is underreported. Additionally, our case highlights the importance of increased vigilance for mucormycosis in patients with prolonged neutropenia prior to HSCT and the potential link of voriconazole prophylaxis and increased risk for mucormycosis.

## 1. Introduction

Infections after hematopoietic stem cell transplant (HSCT) are of particular concern due to their high morbidity and mortality. HSCT places patients at a considerable risk of infection, depending on the severity of immunodeficiency, pathogen exposure, and time since HSCT [1].

One infection with a particularly high mortality is mucormycosis (formerly also zygomycosis), a term used to describe fungal infections caused by filamentous fungi belonging to the order *Mucorales* [2]. On a daily basis, people come in contact with this fungus without being threatened; however, with a weakened immune system, the fungus can become lethal. Mucormycosis invades into the vasculature and causes necrosis and hemorrhage in the organs it attacks [3]. Pulmonary mucormycosis is especially hard to detect as symptoms are similar to bacterial pneumonia [4]. CT scans will sometimes demonstrate halo or reversed halo signs which help support a mucormycosis diagnosis in immunocompromised patients [5]. However, blood and sputum cultures rarely yield any growth. The panfungal *β*-D-glucan test and Aspergillus galactomannan test also do not detect components of the mucoralean cell wall. Therefore, most times the diagnosis can only be made with direct tissue sampling, which in the case of mucormycosis often occurs post-mortem [6].

When undiagnosed, pulmonary mucormycosis invades the pulmonary vasculature then spreads to the heart, mediastinum, and blood. Around 12% of mucormycosis cases occurs after a bone marrow transplant [7]. Mortality rates for pulmonary mucormycosis are around 87%. After undergoing an HSCT, patients with mucormycosis have less than a median 2-month survival rate [3]. Additionally, prophylactic medications given for fungus during HSCT are typically not active against mucormycosis, making prevention less likely [6]. Predisposing risk factors for mucormycosis were highlighted by Kontoyiannis et al. in a previous publication [8].

The above information makes it imperative for providers taking care of HSCT patients to have a high level of suspicion for mucormycosis and initiate early treatment. Here, we present a particularly devastating case to highlight its relatively indolent course until invasion of a vital organ, in this case the brain, occurs. Additionally, we report this case because it is believed mucormycosis in HSCT patients are likely underestimated due to low rates of autopsy [9] and because a high level of suspicion is needed in patients with prolonged neutropenia prior to HSCT. Kontoyiannis et al. state, because of its low incidence, many “hematologists have not accumulated a “critical mass” of experience … to recognize early clinical signs that may be suggestive of invasive mucormycosis” [8]. Lastly, this case highlights a unique sequence of events in that fungal invasion of the frontal lobe led to cerebral edema and ultimately cerebellar tonsillar herniation and death.

## 2. Case Presentation

A 64-year-old woman with a history of refractory acute myeloid leukemia (AML), hypertension, and left ear deafness was admitted for allogeneic bone marrow transplant and subsequently died of intracerebral hemorrhage secondary to mucormycosis 13 days after receiving her stem cells.

### 2.1. Leukemia History

This patient was originally diagnosed with AML 8 months prior to HSCT because of fatigue and pancytopenia on lab work. Peripheral smear at the time showed 43% blasts, and a subsequent bone marrow biopsy showed 60–70% myeloblasts consistent with a diagnosis of AML. Genetic studies done of the bone marrow showed deletions of 16q and 5q, and FLT3 was negative. She was started on induction treatment with 7 + 3 cytarabine/idarubicin which was complicated by neutropenic fever and AML retinopathy.

Neutropenic fever occurred on day 2 of induction treatment admission for which she was given piperacillin/tazobactam until she became afebrile on day 13 of admission. Fevers recurred on day 15 of admission for which cefepime and metronidazole were started. CT scan of her chest was done on day 16, showing a new right lower lobe opacity concerning for pneumonia along with 9 nodules in both the lungs, all less than 6 mm. Treatment dose voriconazole was added for fungal coverage. On day 19, she was broadened to meropenem and anidulafungin due to recurrent fevers. A repeat CT scan of her chest including her sinuses was done showing resolution of her previous consolidation with no evidence of significant sinus disease and no change in her lung nodules. She became afebrile on day 4 on meropenem and anidulafungin but was continued on these antibiotics until day 47 of admission awaiting her absolute neutrophil count (ANC) to reach 500. She was then switched to oral ciprofloxacin, amoxicillin/clavulanic acid, and voriconazole. She completed a total of 7 days of oral antibiotics and then was put on prophylactic voriconazole and acyclovir until HSCT.

After induction, she suffered from prolonged neutropenia and never reached an ANC above 1,130 in the time between the first induction and her death (about 7 months in total). Her treatment after 7 + 3 consisted of cladribine, mitoxantrone, decitabine, and venetoclax with venetoclax finally achieving remission. At the time of transplant, her disease status was in complete remission with incomplete hematologic recovery (CRi) due to sustained cytopenia. On the last office visit prior to being admitted for HSCT, she was noted to have no symptoms and felt overall well.

In terms of further imaging after initial diagnosis, she had a repeat CT scan 7 days prior to HSCT as part of her pretransplant workup to monitor the nodules previously seen. This scan showed stable nodules, all of which did not change in size or appearance. No consolidation or new findings were seen.

### 2.2. Bone Marrow Transplant Course

The patient was admitted and started on trimethoprim-sulfamethoxazole, fluconazole (switched to voriconazole one day prior to HSCT), and valacyclovir prophylaxis. She then started receiving a myeloablative conditioning (MAC) regimen with total body irradiation (TBI) (total dose 12 Gray (Gy) divided into 6 fractions). Two days following TBI completion, she received her donor leukocyte infusion (DLI), followed by cyclophosphamide and mesna 3 days after DLI. She then received her CD34+ stem cells 6 days following DLI infusion. She had her first recorded fever of 101.0 on day (−5) of her transplant, one day following DLI infusion. From this day until the date of death, she was noted to have daily fevers, as high as 104.0. Blood cultures were drawn daily, all of which showed no growth. Sputum cultures were not obtained due to lack of productive cough, and urine cultures showed no growth. She was initially started on cefepime for neutropenic fever which was eventually broadened to meropenem then to piperacillin-tazobactam and vancomycin. She was also switched from prophylactic voriconazole to treatment dose voriconazole 2 days prior to death. Switching to amphotericin B or brain imaging was not considered upon first fevers due to lack of consideration for mucormycosis.

**Figure 1:**
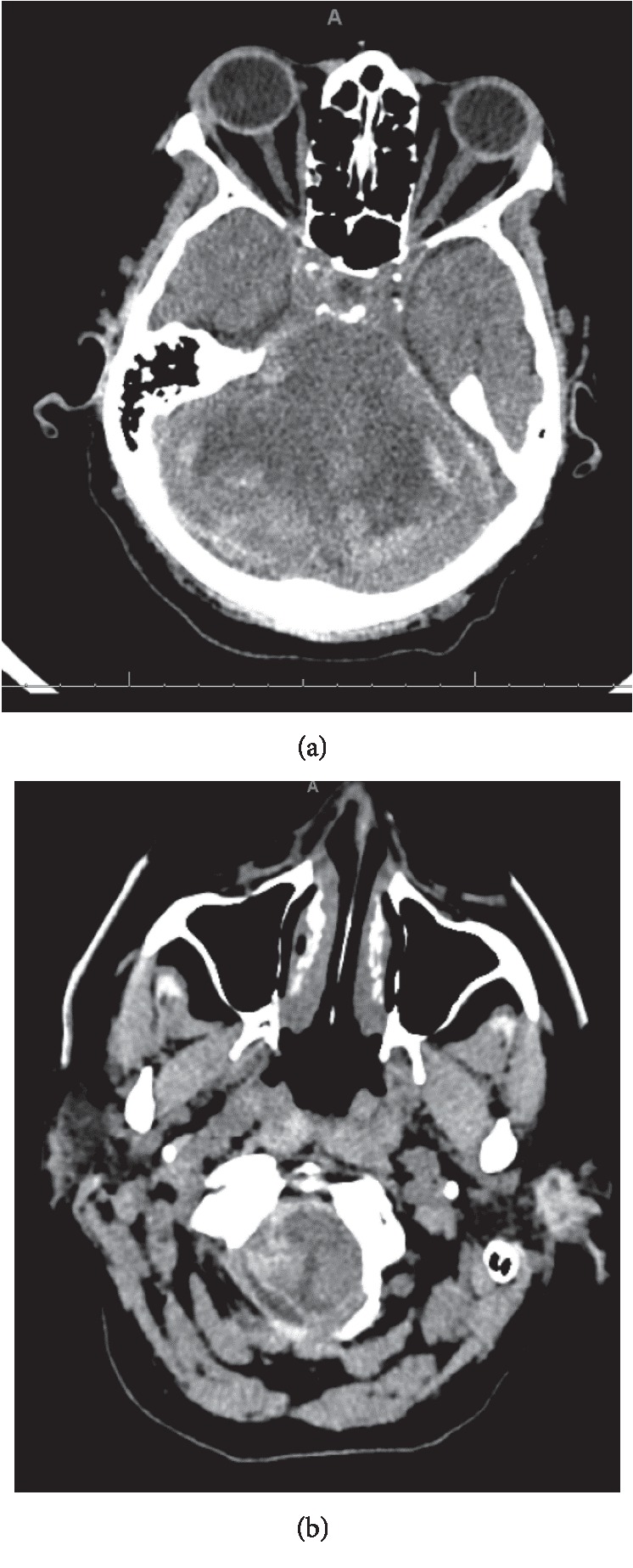
Peripheral hemorrhage in the cerebellar folia with associated areas of hypodensity (a), along with hypodensity and hemorrhage within the brainstem (b), both consistent with ischemia.

**Figure 2:**
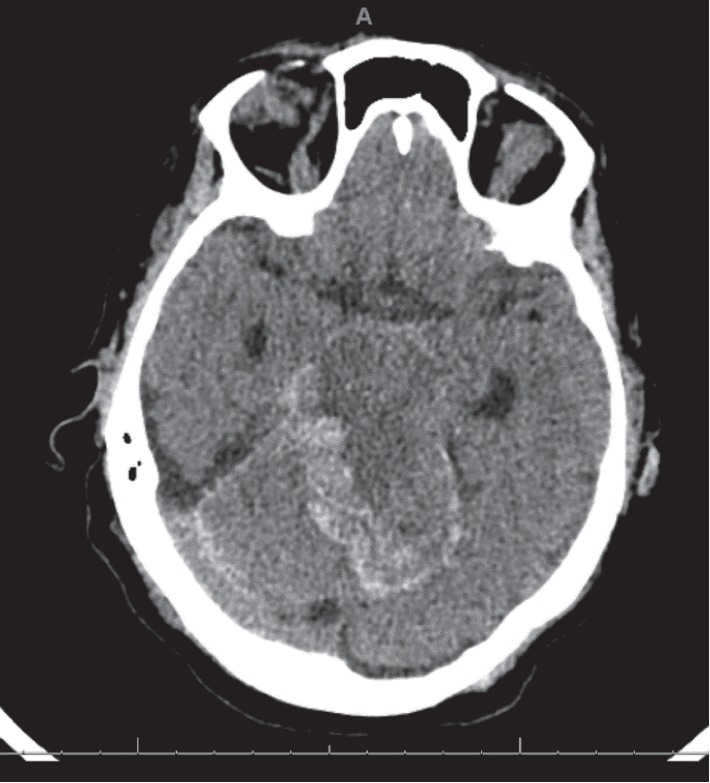
Marked areas of hemorrhage and upward herniation of cerebellum.

Twelve days after receiving her stem cells, she started complaining of severe right shoulder pain; a chest X-ray was obtained which showed a right upper lobe consolidation. CT scan of the chest was done which showed ground glass consolidation with interlobular septal thickening and mucoid impaction. The day after the shoulder pain started, the patient became increasingly fatigue and more lethargic but was answering questions appropriately. Later that afternoon, she was noted by the nursing staff to be completely unresponsive. The patient was emergently intubated and taken for a noncontrast CT scan of the head which showed extensive cerebellar edema with peripheral hemorrhage in the cerebellar folia and petrosal surface of the cerebellum with upward herniation. There was also marked compression of the brainstem and effacement of the fourth ventricle with lucency of the brainstem compatible with ischemia (Figure 1) and associated upward herniation of the cerebellum (Figure 2). Additionally, there was a 7 mm focus of high density in the right frontal lobe and 4 mm focus of hyperdensity in the left frontoparietal junction. About 6 hours after this event, the patient was made comfort care and died shortly thereafter.

### 2.3. Autopsy Reports and Post-Mortem Evaluation

**Figure 3:**
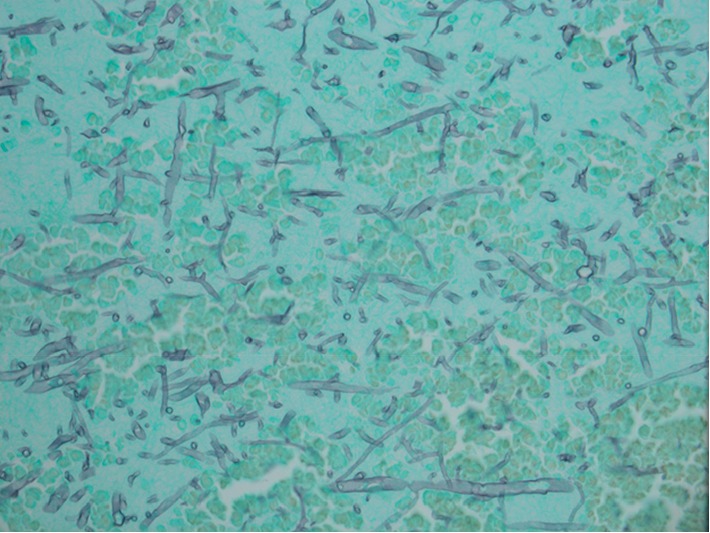
40x right frontal lobe specimen with Gomori methenamine-silver (GMS) staining showing large, nonseptate hyphae with 90 degree angle branching and nonparallel walls consistent with mucormycosis.

**Figure 4:**
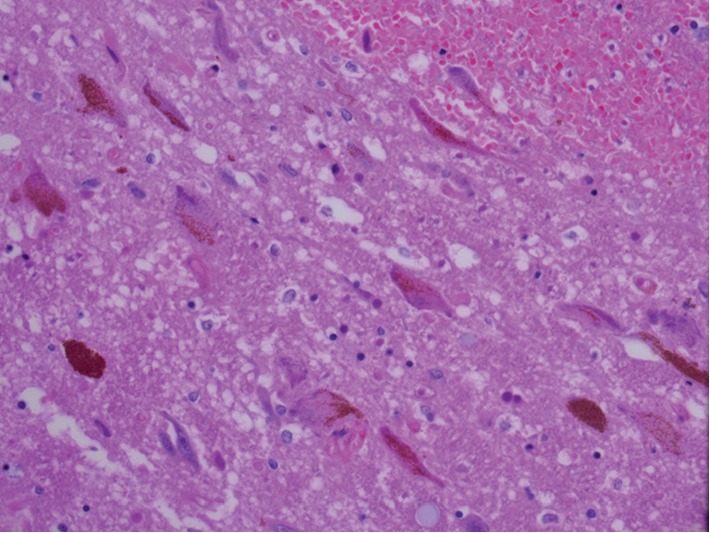
40x pons specimen with H&E staining showing multiple small intraparenchymal pontine hemorrhages consistent with duret hemorrhages.

On autopsy of the brain, it was noted the patient had duret hemorrhage in the pons, cerebellar intraparenchymal hemorrhages and associated subarachnoid hemorrhage with cerebellar edema, right frontal lobe hemorrhagic infarct with angioinvasive fungi, and a small left thalamic hemorrhage. On fungal stain of the right frontal lobe, it was noted she had *Mucor* spp. as can be seen in Figure 3. No polymerase chain reaction (PCR) or fluorescent in situ hybridization (FISH) testing was done due to identifiable appearance on microscopic examination. No fungi were found in the brainstem as can be seen in the cross-sectional imaging of the pons in Figure 4. Acid-fast bacilli (AFB) stains were negative.

Her gross exam of her right lung showed a firm dark-red mass on the right upper lung lobe, measuring 9.5 × 8.0 × 6.8 cm, and the cut surfaces of this mass reveals a well circumscribed, homogenous, round, dark-red fleshy mass resembling a hemorrhagic infarction. On microscopic examination, extensive hemorrhagic areas, pulmonary edema, and small pulmonary infarct areas around some vessels occluded with thrombi were found. Aerobic and anaerobic cultures showed very light growth of vancomycin-resistant *Enterococcus* and very light growth of *Serratia marcescens.* Fungal cultures showed light growth of *Mucor* spp. AFB stains were negative. Fungal staining showed large, nonseptate hyphae with 90 degree angle branching and nonparallel walls consistent with *Mucor* spp. Again, no PCR or FISH testing was done due to identifiable appearance on microscopic examination. Examination of the sinuses showed no significant disease.

The sequence of events most likely leading to her death was concluded to be frontal lobe fungal invasion causing edema and hemorrhage leading to cerebellar tonsillar herniation, brainstem hemorrhage, and ultimately death.

## 3. Discussion

The above case represents a fatal course of mucormycosis following HSCT. Likely, it was our patient's prolonged neutropenia prior to HSCT which put her at a much higher risk for mucormycosis, after obvious colonization. The MAC regimen further suppressed her immune system to a point where dissemination to other organs systems outside of the lung was inevitable. In our search of the literature, only case reports exist of mucormycosis leading to intracerebral or intracerebellar hemorrhage. Takahashi et al. [10] report a case of subcortical hemorrhage while Malik et al. [11] report a case confined to the basal ganglia, whereas Munoz et al. [12] report a lobar hemorrhage case. We could not find any case which describes tonsillar herniation resulting from fungal-related intracerebral swelling. This sequence of events highlights the significant amount of edema invasive mucormycosis can cause, and how quickly it can lead to death. Because lung specimens, both microbiologically and histologically, were positive for *Mucor* spp., this was the likely reservoir for disease dissemination.

One aspect of our case which did not necessarily fit the typical course for mucormycosis after HSCT is the timing of infection. In a study conducted by Xhaard et al., they found infection occurred at a median of 225 days after allogeneic HSCT but with a wide range of 0–2693 days [13]. In a study conducted by Park et al., they found 54.4% of infections from mucormycosis, fusariosis, or scedosporiosis occurred <6 months after HSCT with close to 30% of these occurring 0–2 months after HSCT [14]. Our patient's infection was found about 7 days after HSCT, but likely she simply had undiagnosed indolent infection due to her prolonged neutropenia. This could have accounted for the earlier than “usual” dissemination of infection after her actual HSCT.

The above type of mucormycosis is referred to as disseminated mucormycosis and carries a very high mortality [15]. Current treatment guidelines for disseminated mucormycosis from the European Society of Clinical Microbiology and Infectious Diseases (ESCMID), Fungal Infection Study Group (EFISG), and the European Confederation of Medical Mycology (ECMM) recommend immediate treatment with surgical debridement when appropriate, liposomal amphotericin B (AmB) at a dose of >5 mg/kg or >10 mg/kg with central nervous system involvement (strong recommendation, A IIu). Additionally, their guidelines recommend 800 mg daily of posaconazole as salvage treatment (strong recommendation, A IIu) [16]. Isavuconazole has been approved for use against mucormycosis when amphotericin B is not feasible [16], but is not included yet in the most recent guidelines [17]. Chamilos et al. showed that initiation of therapy within five days after diagnosis of mucormycosis was associated with an improvement in survival as compared with initiation of therapy at six days or more after diagnosis [18]. Additionally, it should be noted, brain lesions in the setting of HSCT are more likely to be caused by mucormycosis rather than aspergillosis and as such aggressive mucormycosis treatment should be initiated promptly once brain lesions are identified [19].

Unfortunately, in our patient, antibiotics were not changed to AmB or an antifungal agent with activity against mucormycosis. Ultimately, given the severity of overall fungal burden, it is unclear whether initiating appropriate antibiotic therapy at pulmonary symptom onset would have halted the progression of disease. Rather, it is imperative to identify patients who have prolonged neutropenia prior to HSCT as being at higher risk for mucormycosis. These patients should be put on posaconazole 200 mg three times daily prophylaxis as per ESMID/EFISG/ECMM guidelines (marginal recommendation, C III). Additionally, their guidelines state that if mucormycosis is a potential differential diagnosis, biopsy should be pursued (strong recommendation, A IIu) [16]. Because our patient had 9 pulmonary nodules on her CT prior to HSCT and had prolonged neutropenia, mucormycosis should have been a consideration and a CT-guided biopsy should have been pursued.

Another important point about our case is our patient's long-term voriconazole prophylaxis prior to HSCT. In a review of voriconazole-associated zygomycosis, it was noted that voriconazole prophylaxis has been associated with increased numbers of mucormycosis. Additionally, they state that voriconazole exposure can modulate the virulence of some *Mucorales*, but the clinical implications of these findings remain unknown [20]. Our patient's voriconazole prophylaxis prior to HSCT may have put her at higher risk for developing mucormycosis and is something practitioners should be vigilant of in preparing patients for HSCT.

The combination of prolonged neutropenia, pulmonary nodules, and long-term voriconazole prophylaxis should have prompted further workup of mucormycosis prior to HSCT. We report this case as a reminder for other practitioners working with HSCT patients to be vigilant of the risk of mucormycosis and to be diagnostically aggressive in order to prevent the outcome we present here.
